# The behavioral, physiological, and biochemical responses of *Lumbriculus variegatus* exposed to cannabidiol and its metabolites

**DOI:** 10.1093/etojnl/vgaf048

**Published:** 2025-02-14

**Authors:** Benjamin S Williams, Georgeena Jomy, Megan Flanagan, Julanta J Carriere, Grace E Labdon, Grace S Hawkes, James McRobbie-Aston, Melisa J Wallace, Claire L Price, Nia A Davies, Aidan Seeley

**Affiliations:** Swansea Worm Integrative Research Laboratory (SWIRL), Swansea University Medical School, Swansea University, Wales, United Kingdom; Swansea Worm Integrative Research Laboratory (SWIRL), Swansea University Medical School, Swansea University, Wales, United Kingdom; Swansea Worm Integrative Research Laboratory (SWIRL), Swansea University Medical School, Swansea University, Wales, United Kingdom; Swansea Worm Integrative Research Laboratory (SWIRL), Swansea University Medical School, Swansea University, Wales, United Kingdom; Swansea Worm Integrative Research Laboratory (SWIRL), Swansea University Medical School, Swansea University, Wales, United Kingdom; Swansea Worm Integrative Research Laboratory (SWIRL), Swansea University Medical School, Swansea University, Wales, United Kingdom; Swansea Worm Integrative Research Laboratory (SWIRL), Swansea University Medical School, Swansea University, Wales, United Kingdom; Swansea Worm Integrative Research Laboratory (SWIRL), Swansea University Medical School, Swansea University, Wales, United Kingdom; Swansea Worm Integrative Research Laboratory (SWIRL), Swansea University Medical School, Swansea University, Wales, United Kingdom; Centre for Cytochrome P450 Biodiversity, Swansea University Medical School, Swansea University, Wales, United Kingdom; Swansea Worm Integrative Research Laboratory (SWIRL), Swansea University Medical School, Swansea University, Wales, United Kingdom; Swansea Worm Integrative Research Laboratory (SWIRL), Swansea University Medical School, Swansea University, Wales, United Kingdom

**Keywords:** *Lumbriculus variegatus*, cannabidiol, behavioral toxicology, invertebrate toxicology, ecotoxicology

## Abstract

Cannabidiol (CBD) is a major non-psychoactive cannabinoid that has been detected in environmental samples, but the ecotoxicological effects remain unknown. In this study, *Lumbriculus variegatus* were exposed to CBD and its metabolites 7-hydroxy-cannabidiol (7-OH-CBD) and 7-carboxy-cannabidiol (7-COOH-CBD). In this study, toxicity, tactile stimulation to elicit stereotypical behaviors, and locomotor activity were measured after 24-hr exposure of *L. variegatus* to CBD and its metabolites. We describe the impacts on dorsal blood vessel pulsation and oxygen consumption after 24-hr exposure to CBD and 7-OH-CBD and the effects on regenerative capacity and total energy reserves after 72 hr of exposure to CBD and 7-OH-CBD. We observed that CBD, 7-OH-CBD, and 7-COOH-CBD displayed toxicity in 50% of test populations at 14.12 µM, 11.29 µM, and 15.36 µM, respectively. A 24-hr exposure to CBD decreased tactile stimulation response to elicit body reversal at ≥ 2.5 µM and helical swimming at ≥ 0.5 µM and reduced locomotor activity. *Lumbriculus variegatus* oxygen consumption was not affected by CBD, but ≥ 2.5 µM significantly reduced dorsal blood vessel pulse rate. We observed that exposure to 7-OH-CBD did not affect the regenerative capacity of *L. variegatus* whereas CBD was shown to reduce regeneration. Exposure to CBD also resulted in a significant decrease in carbohydrates, increased lipids, and no effect on protein levels in *L. variegatus*. We determined that CBD can reduce *L. variegatus* behaviors, decrease pulse rates and regenerative capacity, and disrupt energy reserves. Our findings show that CBD is toxic to this common aquatic organism and the increased availability and use of CBD and related substances warrants further study of their environmental impact.

## Introduction

There is increasing societal interest in the therapeutic use of cannabinoids, a class of chemicals found in plants of the *Cannabis* genus, including *Cannabis sativa, Cannabis indica*, and *Cannabis ruderalis.* Although the psychoactive cannabinoid Δ9-tetrahydrocannabinol (THC) has been a prevailing focus of this research (Leinen et al., 2023; Ujváry & Hanuš, 2016), increasingly, the potential benefits of cannabidiol (CBD) are being proposed. Unlike THC, CBD is a nonpsychoactive cannabinoid that is proposed to be neuroprotective, antiepileptic, anxiolytic, antipsychotic, anti-inflammatory, analgesic, and have anticancerous properties due to its diverse molecular targets (de Almeida & Devi, 2020; Peng et al., 2022). Globally, cannabis is the most commonly abused recreational drug (United Nations Office on Drugs and Crime, 2024), and policies have increased access to medicinal cannabis or cannabis-based products, with the value of the global market expected to reach $46.8 billion in 2025 (Arcview Market Research, 2020). There has also been an increase in direct-to-consumer CBD products, with the United Kingdom CBD market alone valued at $1 billion (Association for the Cannabinoid Industry & The Centre for Medicinal Cannabis, 2021).

Found in groundwater and sewage sludge, humans release cannabinoids into the environment by excretion in feces and in conjugated form in urine (Black et al., 2019; Jurado et al., 2012; Mastroianni et al., 2013). In two studies by Mastroianni et al. (2013) and Black et al. (2019), CBD was detected in 43%–80% of tested sewage sludge samples, compared with 7%–100% of samples containing THC, with CBD concentrations, ranging from 0.1–1.5 µM (Mastroianni et al., 2013). Previous studies have also shown that 0.25–12.7 µM CBD negatively affects aquatic vertebrates (Ahmed et al., 2018; Carty et al., 2018) whereas 0.1–10 µM has significant effects on aquatic invertebrates (Schuel et al., 1987, 1991). Therefore, CBD and its metabolites are contaminants of emerging concern due to limited information on their environmental impacts (How & Gamal El-Din, 2021).

In humans, CBD is metabolized by the cytochrome P450 enzymes, CYP2C9 and CYP2C19, to the metabolite 7-hydroxy-cannabidiol (7-OH-CBD), which is further metabolized to 7-carboxy-cannabidiol (7-COOH-CBD; Beers et al., 2023). While several studies have detected THC and its metabolites in the environment (Black et al., 2019; Mastroianni et al., 2013, 2016; Pandopulos et al., 2022), to date, no study has investigated the metabolites of CBD. However, given the detrimental effects of CBD on aquatic invertebrates (Schuel et al., 1987, 1991) and due to the partition coefficient of CBD (Apul et al., 2020), CBD poses a significant risk to the organisms in close contact with sediments, such as benthic invertebrates.

The annelid *Lumbriculus variegatus* is an endobenthic detritivore species that inhabits shallow freshwater ponds, lakes, and marshes (Drewes, 1999). Detritivores contribute to the ecosystem services of soil formation and nutrient cycling, so detrimental effects on this group will have knock-on effects on these ecosystem services and may therefore affect ecosystem functioning. *Lumbriculus variegatus* is a recommended organism for water and sediment quality evaluation (Organisation for Economic Co-operation and Development, 2007) and has been studied extensively for the impact of pollutants (Aikins et al., 2023; Colombo et al., 2016; O’Gara et al., 2004; Sardo & Soares, 2010; Silva et al., 2021; Vought & Wang, 2018) and increasingly being used to study pharmacologically active compounds (Carriere et al., 2023; Davies et al., 2025; Karlsson et al., 2016; Nentwig, 2007; Seeley et al., 2021, 2024).

Previous studies have quantified the locomotor activity of *L. variegatus* (Davies et al., 2025; Seeley et al., 2021, 2024) and quantified characteristic stereotypical movements of body reversal and helical swimming following stimulation of the anterior or posterior regions, respectively, in these annelid worms (Carriere et al., 2023; Davies et al., 2025; Drewes, 1999; Seeley et al., 2021, 2024), with these behaviors used in environmental studies (O’Gara et al., 2004). Previous studies have also examined the impact of environmental pollutants on dorsal blood vessel (DBV) pulse rates (Wang & Wang, 2021) and energy reserves (Silva et al., 2021). Additionally, asexual reproduction of *L. variegatus* has also been investigated following exposure to environmental contaminants (Aikins et al., 2023). *Lumbriculus variegatus* are also capable of segmental regeneration following injury (Tellez-Garcia et al., 2021; Tweeten & Anderson, 2008), with each fragment capable of regenerating into a fully functional worm (Martinez Acosta et al., 2021).

In this study, we examined *L. variegatus* stereotypical behaviors and locomotor activity following both 10-min and 24-hr exposure to CBD. Furthermore, we measured the behavioral, physiological, and biochemical effects of CBD and its metabolites in *L. variegatus*.

## Material and methods

### *Lumbriculus variegatus* culture

*Lumbriculus variegatus* were procured from Alfa Fish Foods and laboratory‐reared in artificial pond water composed of 1 mM sodium chloride; 13 μM potassium chloride, 4 μM calcium nitrate tetrahydrate; 17 μM magnesium sulfate heptahydrate; 71 μM 4-(2-hydroxyethyl)piperazine-1-ethane-sulfonic acid buffer in UV‐treated deionized water produced by Elix Essential 3 UV Water Purification System (Seeley et al., 2021). Cultured worms were fed TetraMin flakes and 10 mg/L spirulina weekly, maintained at room temperature (18–21 °C), subject to a 16:8‐hr light:dark cycle, and continuous aeration and water filtration using commercial air stones and aquarium filters, respectively. Populations were increased by asexual reproduction for a minimum of 3 months before experimentation to limit colony variation (O’Gara et al., 2004; Seeley et al., 2021). Prior to testing, individual worms were randomly selected, lacked any obvious morphological defects, and ranged from 2–8 cm in length in accordance with previous studies (Carriere et al., 2023; Davies et al., 2025; O’Gara et al., 2004; Seeley et al., 2021, 2024).

### Materials

We purchased (-)-Cannabidiol (#1570) from Bio-Techne (Abingdon, United Kingdom) and dissolved in 100% dimethyl sulfoxide (DMSO) to generate a 50 mM stock solution, aliquoted, and stored at –20 °C. Stock solutions of CBD was further diluted in artificial pond water to give a final DMSO concentration of 0.5% (v/v) and a maximum final concentration of 20 µM. Artificial pond water with 0.5% (v/v) DMSO was used as a vehicle control. The 7-OH-CBD (#C-180) was supplied predissolved in methanol from Sigma-Aldrich (Dorset, United Kingdom) and stored at –20 °C. Stock solutions of 7-OH-CBD were further diluted in artificial pond water to give a final methanol concentration of 0.5% (v/v) and a maximum final concentration of 15 µM. Artificial pond water with 0.5% (v/v) methanol was used as a vehicle control. The 7-COOH-CBD (C-181) was supplied predissolved in methanol by Sigma-Aldrich (Dorset, United Kingdom) and stored at –20 °C. Stock solutions of 7-COOH-CBD were further diluted in artificial pond water to give a final methanol concentration of 0.5% (v/v) and a maximum final concentration of 14 µM. Artificial pond water with 0.5% (v/v) methanol was used as a vehicle control.

### CBD, 7-OH-CBD, and 7-COOH-CBD toxicity assay

*Lumbriculus variegatus* were transferred to a CELLSTAR 6-well plate (Greiner Bio-One) containing artificial pond water only 18–24 hr before experimentation. After this acclimation period, artificial pond water was aspirated and replaced with 0–20 µM CBD, 0–15 µM 7-OH-CBD, 0–14 µM 7-COOH-CBD, or a vehicle control (0.5% [v/v] DMSO in artificial for CBD or 0.5% [v/v] methanol in artificial pond water for 7-OH-CBD and 7-COOH-CBD). After 24 hr of exposure, *L. variegatus* displaying signs of toxicity were recorded. Toxicity was determined by visual inspection for decomposition, determined by partial or complete tissue degeneration and tissue pallor. Data is expressed as a percentage of *L. variegatus* displaying visible toxicity compared with vehicle controls, with three *L. variegatus* per concentration per experimental replicate.

### Measurement of *L. variegatus* stereotypical movement and locomotor activity

The effects of CBD, 7-OH-CBD, or 7-COOH-CBD on the ability of tactile stimulation to elicit stereotypical movement and the effects on locomotor activity of *L. variegatus* were measured as previously described (Seeley et al., 2021). Briefly, *L. variegatus* were acclimatized for a period of 18–24 hr before experimentation by transferring individual *L. variegatus* to each well of a CELLSTAR 6-well plate (Greiner Bio-One) containing 4 ml of artificial pond water at room temperature. After this acclimation period, the pond water was replaced and the baseline ability of the worm to respond to tactile stimulation was tested using a 20–200 μl plastic pipette tip, alternately stimulating the anterior or posterior of the body. The artificial pond water was then removed and immediately replaced with either vehicle control (0.5% [v/v] DMSO or 0.5% [v/v] methanol in artificial pond water), CBD (0–5 µM or 0–20 µM), 7-OH-CBD (0–5 µM) or 7-COOH-CBD (0–5 µM). After a 10-min or 24-hr incubation, the worms were tested again using the same procedure. Following exposure to CBD, 7-OH-CBD, or 7-COOH-CBD, solutions were aspirated from the well and washed to remove any latent residue with fresh artificial pond water. Artificial pond water was immediately aspirated and replaced with fresh artificial pond water. *Lumbriculus variegatus* were then retested 10 min (Recovery [10 min]) and 24 hr (Recovery [24 hr]) after incubation in artificial pond water only. Data are expressed as a ratio of the movement score while in treatment relative to baseline.

To determine the effects on locomotor activity, *L. variegatus* were acclimatized as described above. Following this acclimation period, artificial pond water was replaced with 2 ml fresh artificial pond water to limit movement in the *z*-axis, and baseline locomotor activity was recorded by rapid sequential image collection with a 13-megapixel camera at a rate of one image per second for 50 s. Images were then collected after the immediate replacement of artificial pond water with either vehicle control (0.5% [v/v] DMSO or 0.5% [v/v] methanol in artificial pond water), CBD (0–5 µM or 0–20 µM), 7-OH-CBD (0–5 µM), or 7-COOH-CBD (0–5 µM). Solutions were then removed, the wells washed, and fresh artificial pond water was added. Images were taken after 10 min (Recovery [10 min]) and 24 hr (Recovery [24 hr]) in artificial pond water. Collected images were then analyzed using ImageJ software by superimposing images taken at each time point and using an area of known distance within each image to calibrate ImageJ to pixels per centimeter within each superimposed image set. To determine the area traversed by each worm, the foreground and background were separated using the thresholding functionality of ImageJ to separate the pixels activated by *L*. *variegatus* from those activated by the 6-well plate. The total area covered by the *L*. *variegatus* before exposure, during CBD, 7-OH-CBD, or 7-COOH-CBD exposure, and both recovery time points were then determined based on the calibration of pixels/cm within ImageJ. Data are expressed as a percentage of the locomotor activity by *L*. *variegatus* compared with baseline conditions.

### *Lumbriculus variegatus* DBV pulse rate

The effects of CBD or 7-OH-CBD on the pulse rate of the DBV were conducted using a modified method from Crisp et al. (2010). Individual *L. variegatus* were transferred to CELLSTAR 12-well plates (Greiner Bio-One) containing artificial pond water only and acclimatized for 18–24 hr. Baseline pulse rate measurements were taken by transferring individual *L. variegatus* to a SuperFrost slide, removal of excess liquid, and gentle compression beneath a coverslip to inhibit movement. Recordings were taken using a Nikon SMZ1270i stereomicroscope using NIS-Elements software. The time between two consecutive pulses was determined from a midbody segment to calculate the beats per minute. *Lumbriculus variegatus* were transferred back to the CELLSTAR 12 -well plates (Greiner Bio-One) and exposed to CBD or 7-OH-CBD (0–5 µM) for 24 hr with 0.5% (v/v) DMSO or 0.5% (v/v) methanol in artificial pond water used as a vehicle control, respectively. Pulse rates were then tested 24 hr after exposure to CBD or 7-OH-CBD (0–5 µM). Data are expressed as the change in bpm following CBD or 7-OH-CBD exposure compared to baseline, with three *L. variegatus* per concentration per experimental replicate.

### *Lumbriculus variegatus* oxygen consumption

The effects of CBD or 7-OH-CBD on the oxygen consumption of *L. variegatus* were conducted based on the methods outlined by Tuazon et al. (2022). Ten *L. variegatus* per condition were transferred to a 30 ml specimen bottle 18–24 hr before experimentation and left to acclimatize. After this acclimation period, artificial pond water was aspirated and replaced with 0–5 µM CBD or 7-OH-CBD or vehicle control (0.5% [v/v] DMSO or 0.5% [v/v] methanol in artificial pond water, respectively). A recording of dissolved oxygen (DO_2_) was immediately taken as the baseline (0 hr) using the Jenway 9500 Benchtop Dissolved Oxygen Meter. The DO_2_ was then measured 24 hr after exposure to CBD or 7-OH-CBD (0–5 µM). Data are expressed as a percentage compared with baseline (0 hr) DO_2_.

### Regeneration of *L. variegatus*

To investigate the impact of cannabinoid compounds on the regenerative capacity of *L. variegatus*, individual *L. variegatus* were transferred to CELLSTAR 6-well plates 18–24 hr prior to starting the experiment. After the acclimation period, *L. variegatus* were bisected using dissecting scissors into anterior and posterior regions, with each section returned to a separate well. Following bisection, *L. variegatus* were exposed to 0–5 µM CBD or 7-OH-CBD or a vehicle control (0.5% [v/v] DMSO or 0.5% [v/v] methanol in artificial pond water, respectively). Images of the regenerating tissue from the posterior section (head regeneration) and anterior section (tail regeneration) were taken using the Nikon SMZ1270i stereomicroscope at 0-, 24-, 48- and 72-hr postamputation (HPA), time points used in previous studies of *L. variegatus* regeneration (Tellez-Garcia et al., 2021). Regeneration was quantified using NIS-Elements software to calculate tissue growth in µm^2^ from the site of bisection. Data is expressed as the fold change relative to the tissue at 0 HPA, with three bisected *L. variegatus* per concentration per experimental replicate.

### Analysis of total energy available in *L. variegatus*

To analyze the effect of CBD and 7-OH-CBD on total energy available in *L. variegatus*, we used protocols adapted from Silva et al. (2021) to quantify protein, carbohydrate, and lipid levels. Ten *L. variegatus* per condition were transferred to a 30 ml specimen bottle and acclimatized for 18–24 hr before experimentation. After this acclimation period, artificial pond water was aspirated and replaced with 0–5 µM CBD or 7-OH-CBD or vehicle control (0.5% [v/v] DMSO or 0.5% [v/v] methanol in artificial pond water, respectively) for 72 hr. After 72 hours of exposure to CBD or 7-OH-CBD, *L. variegatus* were removed, dried on filter paper, and weighed on a microbalance. *Lumbriculus variegatus* were then homogenized in 100 μl ice-cold artificial pond water. A 30 μl aliquot of the total homogenate was removed and stored at –80 °C until further lipid quantification. The remaining homogenate was then centrifuged at 16.1 RCF at 4 °C for 15 min. Two 30 μl aliquots of the supernatant were removed and stored at –80°C for protein and carbohydrate quantification.

Protein quantification was conducted using the Bradford method (Bradford, 1976) using bovine serum albumin as the standard. The absorbance was measured in triplicate at 595 nm using the FLUOstar Omega Microplate Reader (BMG Labtech).

We used the Dubois method for carbohydrate quantification (DuBois et al., 1956) using glucose as the standard. Samples were incubated with phenol (5%) and sulfuric acid (≥ 98%) for 10 min. Each sample was vortexed, incubated at 90 °C for 5 min, removed, and incubated at room temperature for a further 5 min. The absorbance was then read in triplicate using the FLUOstar Omega Microplate Reader (BMG Labtech) at 492 nm.

The Bligh and Dyer (1959) method was utilized for lipid extraction with extraction conducted by resuspension of samples in 300 µl deionized water with samples then transferred to 2 ml glass gas chromatography (GC) vials. 500 µl chloroform (119.37 M; >99%) and 500 µl methanol were added to the samples (32.04 M; for high performance liquid chromatography; > 95%) and the organic phase of each sample was then transferred to a clean glass GC vial and dried to complete dryness using a SpeedyVac. Lipid quantification was conducted using a modified protocol from Men et al. (2019). A total of 150 µl sulfuric acid (≥ 98%) was added to each sample and incubated for 20 min at 90 °C. Then, 450 µl of phospho-vanillin reagent was added to the samples and samples were left at room temperature for 10 min. Absorbance was then read in triplicate using the FLUOstar Omega Microplate Reader (BMG Labtech) at 530 nm. Triolein was used as the standard for quantification.

Absorbances were converted into energetic values of the fractions of energy available using the corresponding energy of combustion obtained from De Coen and Janssen (1997); 24,000 mJ/mg protein; 17,500 mJ/mg glycogen; and 39,500 mJ/mg lipid. Data is expressed as mJ/mg organism and the total energy available (E_a_) was calculated as the sum of energy from proteins (E_Protein_), Energy from carbohydrates (E_Carbohydrate_) and energy from lipids (E_Lipid_; expressed as mJ/mg organism).

### Statistical analysis

The sample size for each assay and treatment was ≥ 3 experimental repeats. Data are displayed as the mean ± SEM for each data set. Statistical analysis was performed in GraphPad Prism 10 with *p < *0.05 as the threshold for statistical significance.

## Results

### Toxicity and behavioral effects of 24-hr exposure to CBD and its metabolites 7-OH-CBD & 7-COOH-CBD

**Figure 1.**
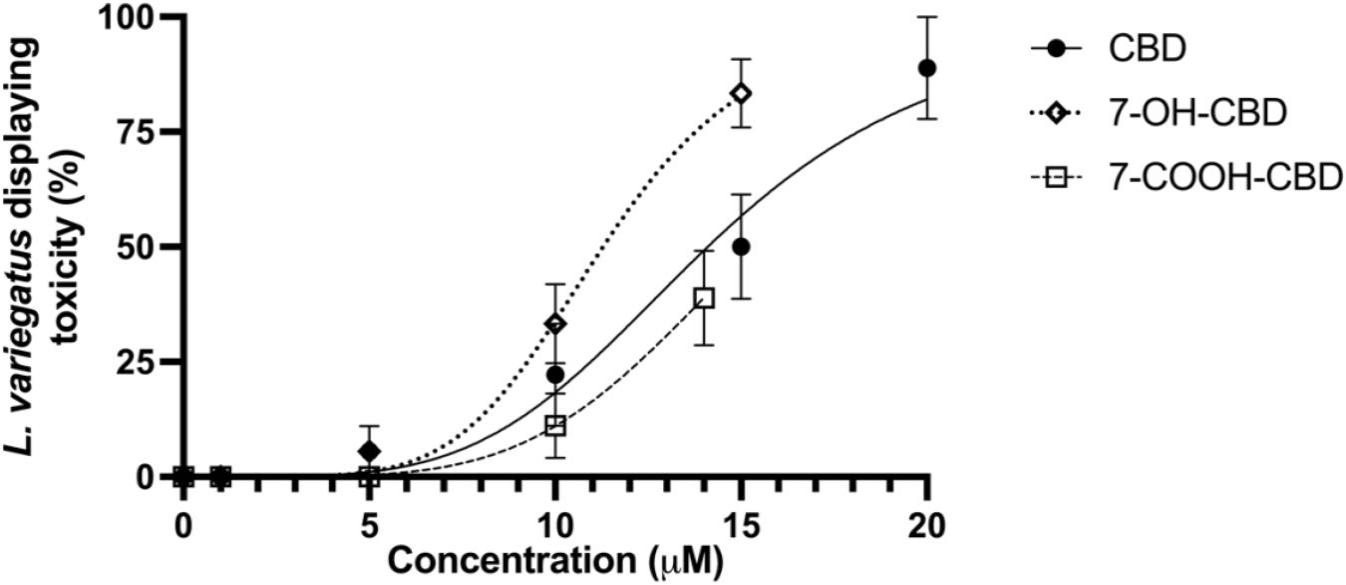
Toxicity of 24-hr exposure to cannabidiol (CBD) and CBD metabolites in *Lumbriculus variegatus. Lumbriculus variegatus* was exposed to CBD (0–20 µM), 7-hydroxy-cannabidiol (0–15 µM), or 7-carboxy-cannabidiol for 24 hr to observe signs of whole organism toxicity. After 24 hr of exposure, worms displaying decomposition, as determined by visible partial or complete tissue degeneration and whole-organism tissue pallor, were counted. Data are expressed as a percentage of *L. variegatus* displaying toxicity. Error bars represent the SEM, *n *=* *6 with three worms per condition per replicate. 7-OH-CBD = 7-hydroxy-cannabidiol; 7-COOH-CBD = 7-carboxy-cannabidiol.

In *L. variegatus* exposed to CBD for 24 hr, we observed that the lowest observed adverse effect level (LOAEL) was 5 µM, where 5.55 ± 5.55% of *L. variegatus* displayed signs of toxicity (Figure 1, *n *=* *6). At the highest tested concentration of 20 µM CBD, 88.88 ± 11.12% displayed signs of toxicity when exposed (Figure 1, *n *=* *6). The no observed adverse effect level (NOAEL) for 24-hr exposure to CBD was determined to be 1.0 µM CBD, where no *L. variegatus* displayed signs of toxicity (Figure 1, *n *=* *6). Moreover, we determined that 14.12 µM (95% confidence interval [CI]: 12.28–15.90 µM) CBD would be sufficient to cause toxicity in 50% of *L. variegatus* test populations.

Similarly, *L. variegatus* exposed to 7-OH-CBD for 24 hr had a LOAEL of 5.0 µM and the highest tested concentration, 15 µM 7-OH-CBD, caused toxicity in 83.35 ± 7.45% of test populations (Figure 1, *n *=* *6). The NOAEL for 7-OH-CBD was equimolar to CBD at 1.0 µM. We observed an increased toxic effect of 7-OH-CBD compared with parental CBD, with 11.29 µM (95% CI: 10.53–12.09 µM) 7-OH-CBD sufficient to cause toxicity in 50% of *L. variegatus* test populations. However, 7-COOH-CBD had reduced toxicity compared with CBD and 7-OH-CBD, with a NOAEL of 5 µM and a LOAEL of 10 µM. The highest tested concentration, 14 µM 7-COOH-CBD, caused toxicity in 38.88 ± 10.25% of test populations (Figure 1, *n *=* *6) with 15.36 µM (95% CI: 14.03–19.94 µM) predicted to cause toxicity in 50% of *L. variegatus* test populations.

**Figure 2.**
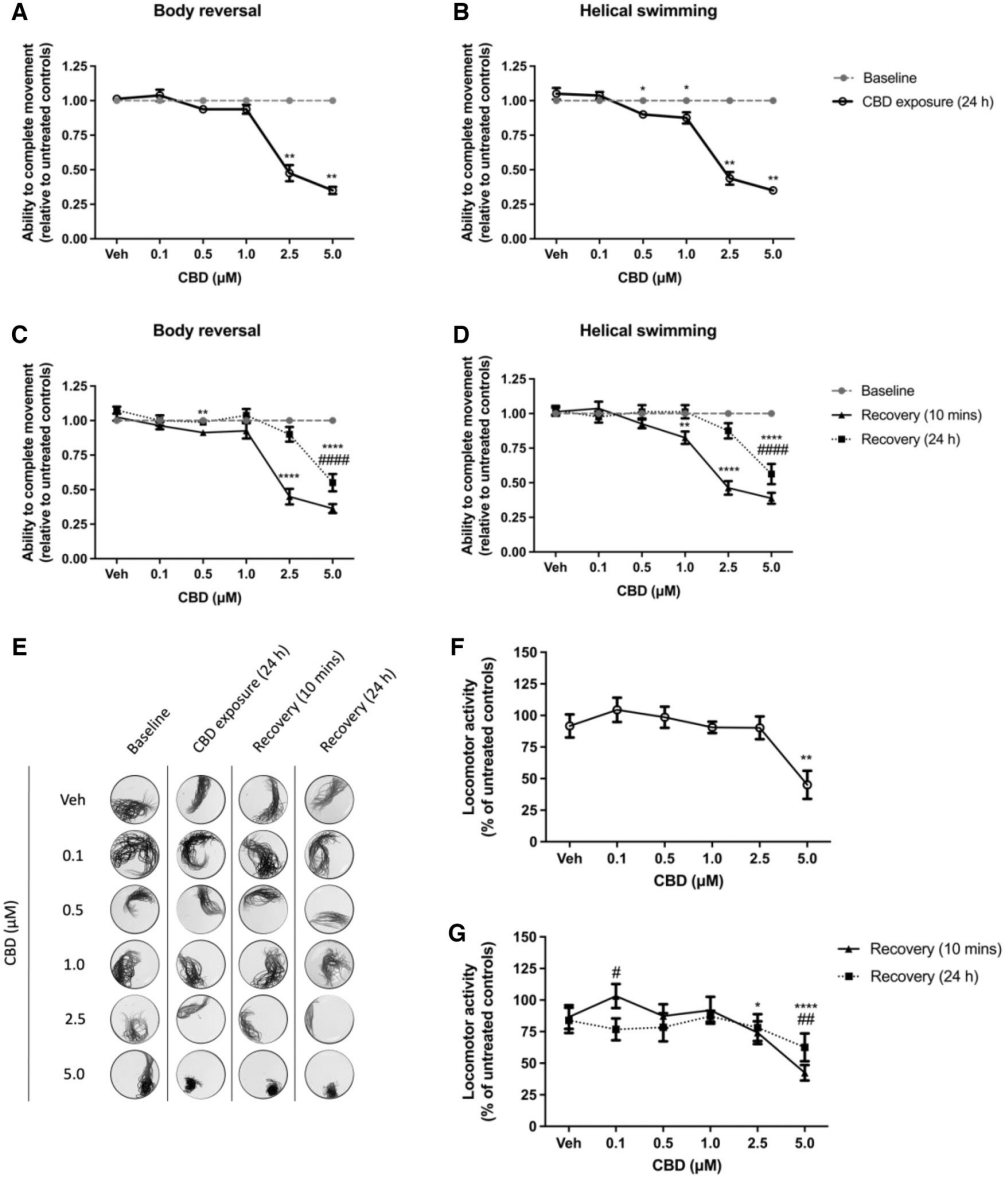
The effect of 24-hr exposure to 0–5 µM cannabidiol (CBD) on *Lumbriculus variegatus* behavior. *Lumbriculus variegatus* were exposed to CBD (0–5 µM) for 24 hr and tested for the ability of tactile stimulation to elicit (A) body reversal or (B) helical swimming. Following removal of CBD, the ability of *L. variegatus* to perform (C) body reversal or (D) helical swimming was tested after 10 min and 24 hr. Data are expressed as a ratio of the movement score after exposure relative to the movement score at baseline. (E) Representative superimposed images analyzed in ImageJ showing the effect of 24 hr of exposure to CBD on locomotor activity measured before CBD exposure (Baseline), after 24-hr exposure to 0–5 µM CBD (CBD Exposure [24 hr]), 10 min after CBD removal (Recovery [10 min]) and 24 hr after CBD removal (Recovery [24 hr]). Quantification of the area covered by *L. variegatus* following (F) 24 hr of exposure to 0–5 µM CBD and (G) removal of CBD for 10 min (Recovery [10 min]) and 24 hr (Recovery [24 hr]), expressed as a percentage of the locomotor activity at baseline. Analyses were conducted by comparing CBD exposure conditions with baseline conditions by paired nonparametric two-tailed *t*-test for stereotypical movement assays and paired parametric two-tailed *t*-test for locomotor activity. A two-way analysis of variance with Dunnett’s posttest was used to analyze 10-min and 24-hr recovery time points compared with baseline conditions for *L. variegatus*. */# *p < *0.05, **/## *p < *0.01, ****/#### *p < *0.0001; where * refers to statistical significance between Baseline and CBD Exposure (24 hr) or statistical significance between Baseline and Recovery (10 min), # refers to statistical significance between Baseline and Recovery (24 hr). Error bars represent the SEM, *n *=* *8 with a single *L. variegatus* exposed to each concentration. Veh = 0.5% (v/v) dimethyl sulfoxide in artificial pond water.

We observed the effects of 24-hr exposure to CBD on the ability of *L. variegatus* to respond to tactile stimulation and its effect on *L. variegatus* locomotor activity. Exposure to ≤ 5 µM CBD for 24 hr was sufficient to elicit significant effects on *L. variegatus* tactile stimulation response (Figure 2A–D, *n *=* *8). After 24 hr of exposure to ≥ 2.5 µM CBD, *L. variegatus* displayed a reduced capacity for tactile stimulation to elicit body reversal behaviors (*p < *0.01, Figure 2A, *n *=* *8) and at ≥ 0.5 µM CBD, a reduced capacity for tactile stimulation to elicit helical swimming behaviors (*p < *0.05, Figure 2B, *n *=* *8). Removal of CBD and incubation in artificial pond water for 10 min was insufficient to reverse these effects (*p < *0.05, Figure 2C, D, *n *=* *8). However, after 24 hr of incubation in artificial pond water, *L. variegatus* response to tactile stimulation to elicit body reversal and helical swimming recovered at ≤ 2.5 µM (*p > *0.05, Figure 2C, D, *n *=* *8) with movements only inhibited at 5 µM (*p < *0.0001, Figure 2C, D, *n *=* *8). Conversely, 24-hr exposure to ≤ 2.5 µM CBD did not affect locomotor activity of *L. variegatus* (*p > *0.05, Figure 2E, F, *n *=* *8). However, 5 µM CBD produced a significant hypokinetic effect in *L. variegatus*, with locomotor activity reduced by 54.88 ± 11.23% (*p = *0.0018, Figure 2F, *n *=* *8).

We determined that removal of CBD and incubation in artificial pond water for 10 min resulted in significant hypokinetic effects in *L. variegatus* exposed to 2.5 and 5 µM CBD, with locomotory activity reduced by 25.80 ± 9.06% (*p = *0.038, Figure 2G, *n *=* *8) and 57.59 ± 6.17% (*p < *0.0001, Figure 2G, *n *=* *8), respectively. Interestingly, we observed hypokinetic effects on locomotor activity after 24 hr of incubation in artificial pond water following 0.1 µM CBD exposure, where locomotor activity was reduced by 23.18 ± 8.60% (*p = *0.038, Figure 2G, *n *=* *8). Further, we observed a recovery of locomotor activity in *L. variegatus* exposed to 2.5 µM, with locomotor activity being indistinguishable from baseline conditions (*p > *0.05, Figure 2G, *n *=* *8), whereas locomotor activity in *L. variegatus* exposed to 5 µM CBD remained reduced (*p < *0.0001, Figure 2G, *n *=* *8).

We also determined the effects of short-term exposure to 0–20 µM CBD for 10 min was sufficient to elicit significant effects on both tactile stimulation response and locomotor behaviors of *L. variegatus* (see online supplementary material Figure S1).

After 10 min of exposure to ≥ 5 µM CBD, *L. variegatus* displayed a reduced capacity for body reversal and helical swimming movements in response to tactile stimulation (*p < *0.05, see online supplementary material Figure S1A, B, *n *=* *8). Removal of CBD and incubation in artificial pond water for 10 min was insufficient to reverse these effects (*p < *0.05, see online supplementary material Figure S1C, D, *n *=* *8). However, after 24 hr of recovery in artificial pond water, *L. variegatus* response to tactile stimulation to elicit body reversal recovered at 5–15 µM with movements only inhibited at 20 µM (*p < *0.05, see online supplementary material Figure S1C, D, *n *=* *8).

Conversely, 10-min exposure to 0–20 µM CBD did not affect locomotor activity of *L. variegatus* (*p > *0.05, see online supplementary material Figure S1E, F, *n *=* *8) but exposure to 5 µM CBD and incubation in artificial pond water for 10 min produced hyperkinetic effects on locomotory activity, with *L. variegatus* movement increasing by 26.60 ± 8.68% (*p *=* *0.02, see online supplementary material Figure S1G, *n *=* *8). Following 24 hr of recovery in artificial pond water, 15 µM and 20 µM CBD was shown to have hypokinetic effects in *L. variegatus*, with movement reduced by 29.58 ± 11.64% and 39.22 ± 13.52%, respectively (*p < *0.05, see online supplementary material Figure S1G, *n *=* *8).

**Figure 3.**
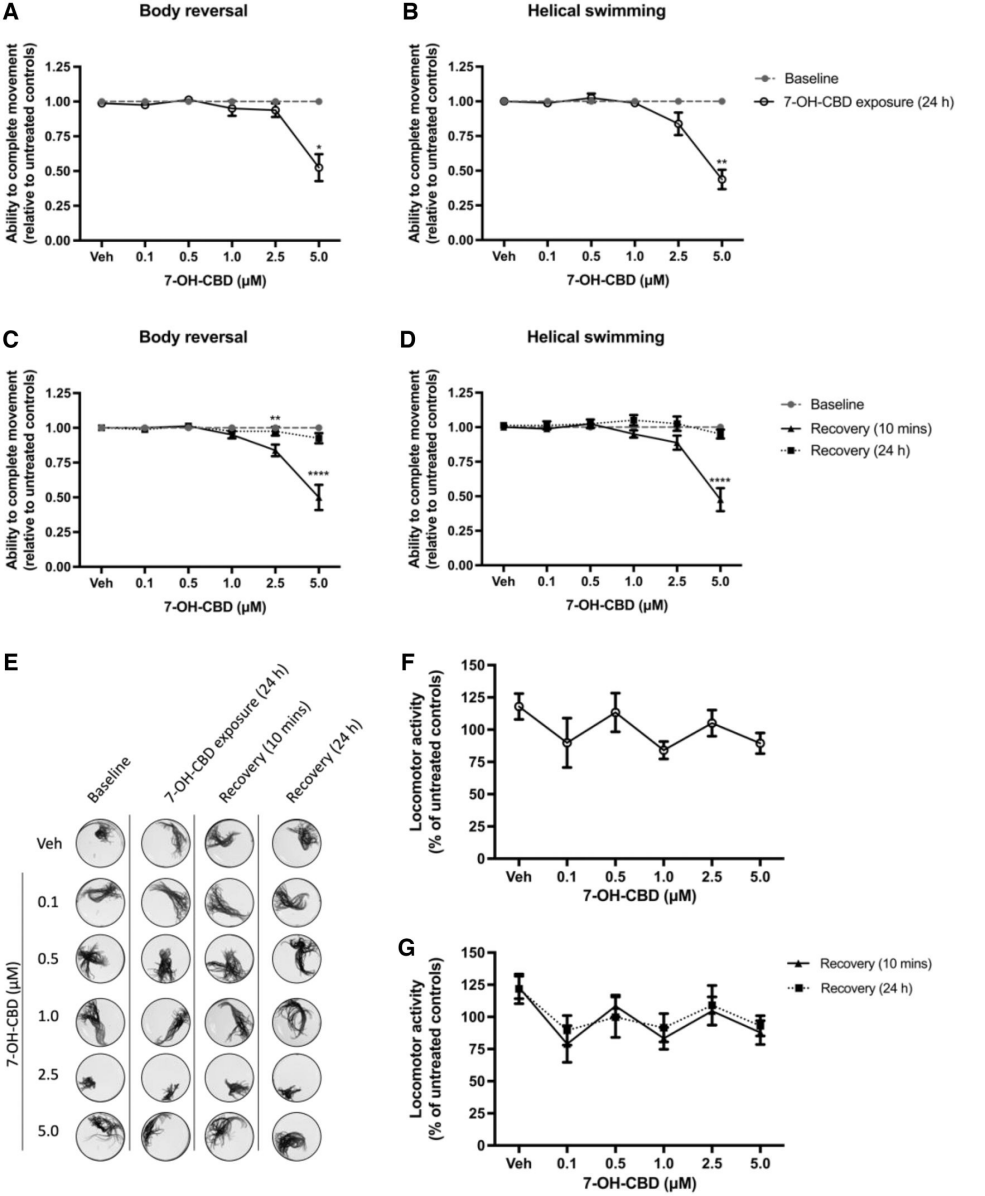
The effect of 24-hr exposure to 0–5 µM 7-hydroxy-cannabidiol (7-OH-CBD) on *Lumbriculus variegatus* behavior. *Lumbriculus variegatus* were exposed to 7-OH-CBD (0–5 µM) for 24 hr and tested for the ability of tactile stimulation to elicit (A) body reversal or (B) helical swimming. Following removal of 7-OH-CBD, the ability of *L. variegatus* to perform (C) body reversal or (D) helical swimming was tested after 10 min and 24 hr. Data are expressed as a ratio of the movement score after exposure relative to the movement score at baseline. (E) Representative superimposed images analyzed in ImageJ showing the effect of 24 hr of exposure to 7-OH-CBD on locomotor activity measured before 7-OH-CBD exposure (Baseline), after 24 hr of exposure to 0–5 µM 7-OH-CBD (7-OH-CBD Treatment [24 hr]), 10 min after 7-OH-CBD removal (Recovery [10 min]) and 24 hr after 7-OH-CBD removal (Recovery [24 hr]). Quantification of the area covered by *L. variegatus* following (F) 10 min of exposure to 0–5 µM 7-OH-CBD and (G) removal of 7-OH-CBD for 10 min and 24 hr are expressed as a percentage of the locomotor activity at baseline. Analyses were conducted by comparing 7-OH-CBD exposure conditions with baseline conditions by paired nonparametric two-tailed *t*-test for stereotypical movement assays and paired parametric two-tailed *t*-test for locomotor activity. A two-way analysis of variance with Dunnett’s posttest was used to analyze 10-min and 24-hr recovery time points compared with baseline conditions for *L. variegatus*. *p < 0.05, **p < 0.01, ****p < 0.0001. No statistical significance was observed between Baseline and Recovery (24 hr). Error bars represent the SEM, *n *=* *8 with a single *L. variegatus* exposed to each concentration. Veh = 0.5% (v/v) methanol in artificial pond water.

When *L. variegatus* were exposed to concentrations of 0–5 µM 7-OH-CBD for 24 hr, we observed that ≤ 2.5 µM did not affect tactile stimulation to elicit body reversal or helical swimming behaviors (*p > *0.05, Figure 3A, B, *n *=* *8), with only 5 µM 7-OH-CBD significantly inhibiting these behaviors (*p < *0.05, Figure 3A, B, *n *=* *8). Removal from 7-OH-CBD and incubation in artificial pond water for 10 min showed inhibition of body reversal following tactile stimulation at 2.5 µM (*p = *0.008, Figure 3C, *n *=* *8) with both movements inhibited at 5 µM 7-OH-CBD (*p < *0.0001, Figure 3C, D, *n *=* *8). However, we observed that following 24 hr in artificial pond water, the ability of tactile stimulation to elicit both behaviors fully recovered, with responses indistinguishable from baseline conditions (*p > *0.05, Figure 3C, D, *n *=* *8). Furthermore, we observed no effect on locomotor activity of *L. variegatus* exposed to 0–5 µM 7-OH-CBD or after removal and incubation in artificial pond water (*p > *0.05, Figure 3E–G, *n *=* *8).

7-Carboxy-cannabidiol had less effect on tactile stimulation to elicit stereotypical movements and locomotor activity when compared with CBD and 7-OH-CBD. Exposure to 0–5 µM 7-COOH-CBD for 24 hr, after the removal of 7-COOH-CBD and incubation in artificial pond water, did not affect the ability of *L. variegatus* to respond to tactile stimulation (*p > *0.05, see online supplementary material Figure S2A–D, *n *=* *8). Furthermore, 7-COOH-CBD had minimal effects on locomotor activity, with only 0.1 µM of 7-COOH-CBD observed to cause a 21.52 ± 6.77% reduction in locomotor activity (*p *=* *0.016, see online supplementary material Figure S2F, *n *=* *8), with no effects on locomotor activity observed at ≥ 0.5 µM (*p > *0.05, see online supplementary material Figure S2F, *n *=* *8). Following 24 hr of recovery in artificial pond water, 0.5 µM was shown to have a hypokinetic effect in *L. variegatus*, with movement reduced by 29.06 ± 10.95% (*p = *0.03, see online supplementary material Figure S2F, *n *=* *8). No other effects on locomotor activity were observed for any other test concentration or timepoint (*p > *0.05, see online supplementary material Figure S2F, *n *=* *8).

### The effects of CBD and 7-OH-CBD on DBV pulsations and oxygen consumption

Having found that CBD and 7-OH-CBD had significant effects on *L. variegatus* behavior, we then investigated the physiological impact on *L. variegatus* DBV pulse rates and oxygen consumption by measurement of DO_2_.

**Figure 4.**
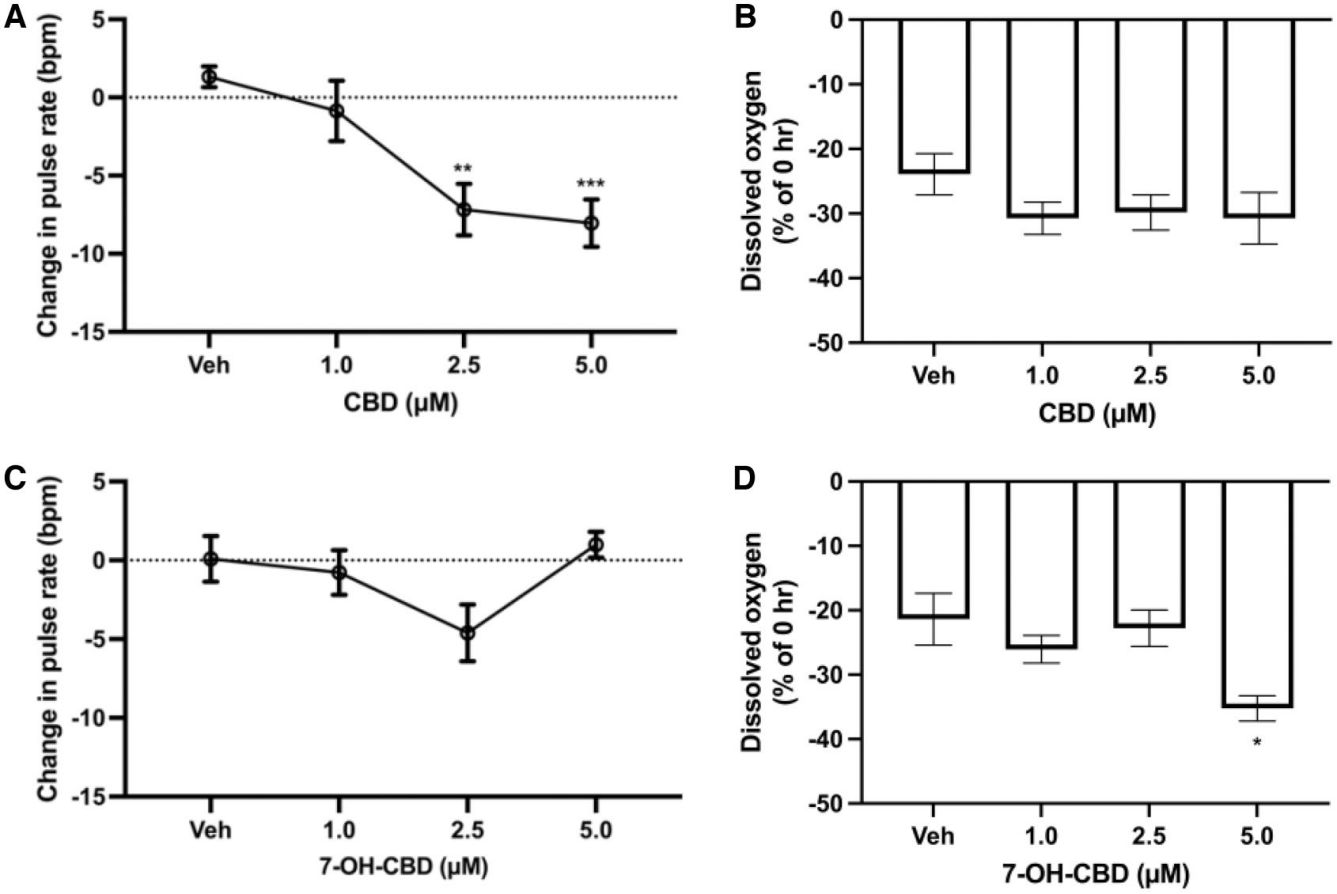
The effect on *Lumbriculus variegatus* dorsal blood vessel pulse rate and oxygen consumption during 24-hr exposure to 0–5 µM cannabidiol (CBD) or 7-hydroxy-cannabidiol (7-OH-CBD). (A) Effects on dorsal blood vessel pulsation rates in *L. variegatus* exposed to 0–5 µM CBD. The horizontal line at 0 indicates the average baseline pulsation rate of 12 bpm. **p < 0.01, ***p < 0.001. *n *≥* *7 with a single *L. variegatus* exposed to each concentration. (B) Dissolved oxygen was measured as a percentage change from 0 hr after exposure to 0–5 µM CBD. *n *=* *3 with 10 worms per condition per replicate. (C) Effects on dorsal blood vessel pulsation rates in *L. variegatus* exposed to 0–5 µM 7-OH-CBD. The horizontal line at 0 indicates the average baseline pulsation rate of 12 bpm. No significant difference was observed in 7-OH-CBD exposure. *n ≥ *7 with a single *L. variegatus* exposed to each concentration. (D) Dissolved oxygen was measured as a percentage change of 0 hr after exposure to 0–5 µM 7-OH-CBD; *n *=* *3 with ten worms per condition per replicate. Error bars represent the SEM. bpm = beats per minute, Veh = 0.5% (v/v) dimethyl sulfoxide in artificial pond water for CBD or 0.5% (v/v) methanol in artificial pond water for 7-OH-CBD.

*Lumbriculus variegatus* exposed to 0–5 µM CBD demonstrated a significant decrease in the pulse rate at 2.5 µM and 5 µM of 7.17 ± 1.65 and 8.04 ± 1.51 bpm, respectively (*p < *0.01, Figure 4A, *n *≥* *7). Cannabidiol exposure demonstrated no significant effect on oxygen consumption (*p > *0.05, Figure 4B, *n *=* *3). Conversely, 7-OH-CBD exposure had no significant effects on the DBV pulse rate of *L. variegatus* (*p > *0.05, Figure 4C, *n *≥* *7); however, there was a significant increase in *L. variegatus* oxygen consumption demonstrated by the 35.22 ± 1.97% decrease in DO_2_ (*p = *0.034, Figure 4D, *n *=* *3).

### Effects of CBD and 7-OH-CBD exposure on regeneration

*Lumbriculus variegatus* are capable of segmental regeneration when injured, with fragments capable of regenerating into new individuals. To measure the impact of CBD and 7-OH-CBD on this process, we investigated the regenerative capacity of *L. variegatus* following bisection.

**Figure 5.**
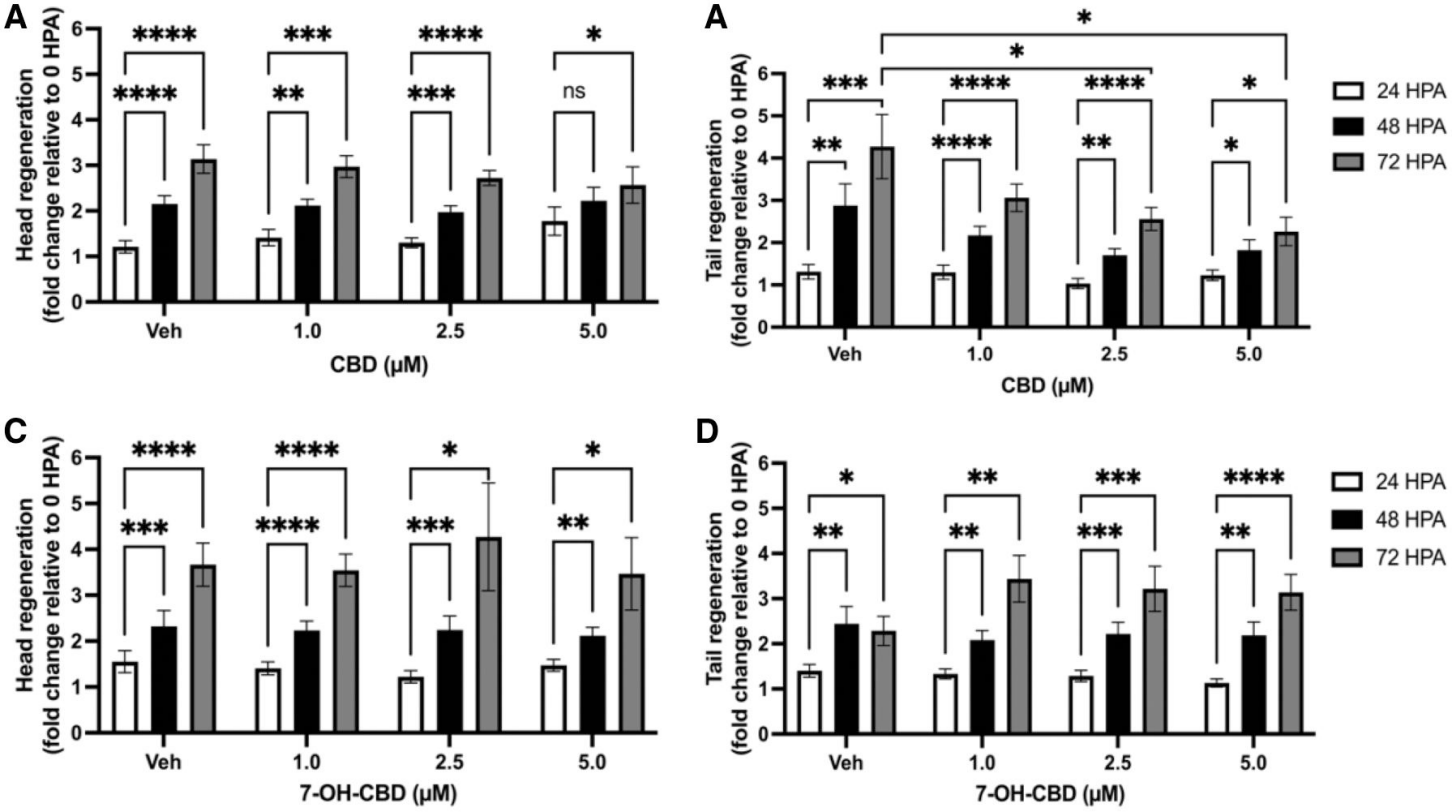
Effects of 0–5 µM cannabidiol (CBD) and 7-hydroxy-cannabidiol (7-OH-CBD) on *Lumbriculus variegatus* regeneration. Following bisection, *L. variegatus* were exposed to 0–5 µM CBD or 7-OH-CBD and regenerative capacity was assessed at 0-, 24-, 48- and 72-hr postamputation (HPA). Regeneration over 72 hr of *L. variegatus* (A) head and (B) tail when exposed to 0–5 µM CBD. (C) Head and (D) tail regeneration when exposed to 0–5 µM 7-OH-CBD. Data are expressed as fold change relative to 0 HPA. Analysis was conducted by two-way analysis of variance with Dunnett’s posttest. *n *≥* *17 for each concentration. Error bars represent the SEM. Veh = 0.5% (v/v) dimethyl sulfoxide in artificial pond water for CBD or 0.5% (v/v) methanol in artificial pond water for 7-OH-CBD.

As seen in Figure 5A, we observed significant regeneration of the head tissue occurred between 24- and 48-hr HPA at ≤2.5 µM CBD (*p < *0.01, *n *≥* *17). Conversely, 5 µM CBD did not show significant increases in tissue regeneration during this time (*p > *0.05, Figure 5A, *n *≥* *17). By 72 HPA, all CBD exposures exhibited a significant increase in head regeneration between 24 and 72 HPA (*p < *0.05, Figure 5A, *n *≥* *17). Moreover, no significant differences in head regeneration were observed between any CBD concentrations at any of the time points tested (*p > *0.05, Figure 5A, *n *≥* *17). Conversely, we determined significant tail regeneration at 48 and 72 HPA compared with 24 HPA for all CBD exposures (*p < *0.05, Figure 5B, *n *≥* *17) but tail regeneration at 2.5 µM and 5 µM was significantly reduced at 72 HPA compared with the tail regeneration observed in vehicle controls (*p < *0.05, Figure 5B, *n *≥* *17).

Additionally, we determined no significant difference in *L. variegatus* head or tail regeneration when exposed to 1–5 µM 7-OH-CBD compared with regeneration in vehicle controls, but that all exposures exhibited significant regenerative capacity of head and tail tissues at 48 and 72 HPA (*p < *0.05, Figure 5C, D, *n *≥* *17).

### Effect of CBD and 7-OH-CBD exposure on total energy available

**Figure 6.**
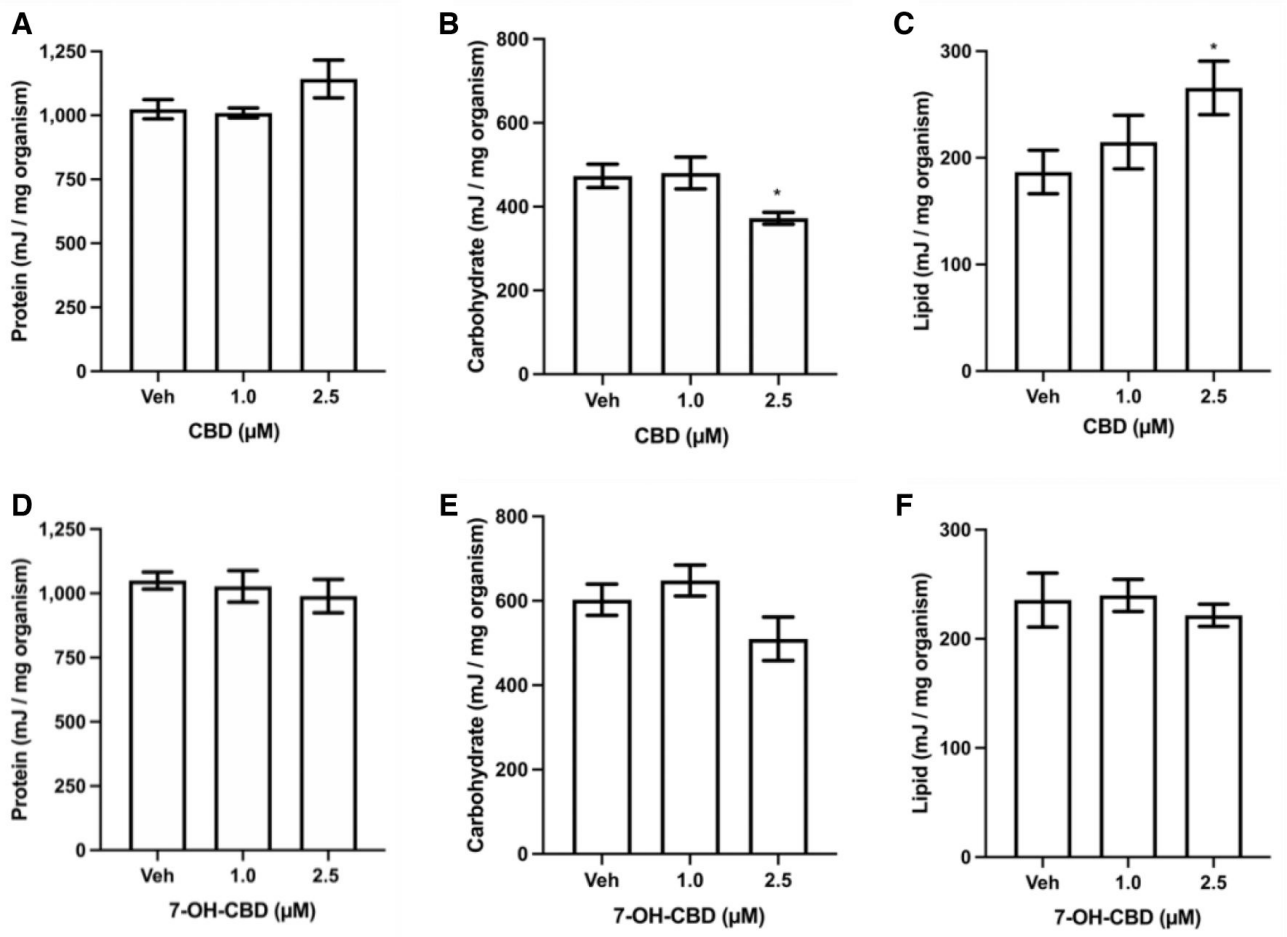
Quantification of protein, carbohydrate and lipid content of *Lumbriculus variegatus* after exposure to cannabidiol (CBD) and 7-hydroxy-cannabidiol (7-OH-CBD). Levels of energy reserves of (A) protein, (B) carbohydrates, and (C) lipids when *L. variegatus* were exposed to 0–2.5 µM CBD for 72 hr; *n *≥* *4 with 10 *L. variegatus* per replicate measured in triplicate for each concentration. (D) protein, (E) carbohydrates, and (F) lipid energy reserves in *L. variegatus* after exposure to 0–2.5 µM 7-OH-CBD for 72 hr; *n *≥* *5 with 10 *L. variegatus* per replicate measured in triplicate for each concentration. * *p < *0.05. Analyses were conducted by comparing CBD or 7-OH-CBD exposure with Veh by a two-tailed *t*-test. Error bars represent the SEM. Veh = 0.5% (v/v) dimethyl sulfoxide in artificial pond water for CBD or 0.5% (v/v) methanol in artificial pond water for 7-OH-CBD.

Finally, we measured the available energy in *L. variegatus* by quantification of protein, carbohydrates, and lipids following exposure to CBD or 7-OH-CBD for 72 hr. We observed no significant difference in protein levels within whole *L. variegatus* homogenate exposure to ≤2.5 µM CBD (*p > *0.05, Figure 6A, *n *≥* *4). However, we determined a significant decrease in carbohydrate levels (*p = *0.025, Figure 6B, *n *≥* *4) and an increase in lipid levels (*p = *0.04, Figure 6C, *n *≥* *4) when exposed to 2.5 µM CBD. However, we observed no change in total E_a_ when *L. variegatus* were exposed to CBD compared with vehicle controls (*p > *0.05, Table 1, *n *≥* *4). When exposed to equimolar concentrations of 7-OH-CBD, we determined there was no difference in protein, carbohydrate, or lipid levels (*p > *0.05, Figure 6E, F, n ≥ 5), or E_a_ (*p > *0.05, Table 1, *n *≥* *5), in whole *L. variegatus* homogenate.

**Table 1.** Total energy available (E_a_) of *Lumbriculus variegatus* after 72-hr exposure to cannabidiol (CBD) or 7-hydroxy-cannabidiol (7-OH-CBD).

Compound	Concentration	E_a_ (E_Protein_ + E_Carbohydrate_ + E_Lipid_)
**CBD**	Veh	1684 ± 49
	1.0 µM	1704 ± 29
	2.5 µM	1780 ± 103
**7-OH-CBD**	Veh	2161 ± 246
	1.0 µM	1905 ± 90
	2.5 µM	1722 ± 83

*Note.* The total energy available, E_a_, is the sum of E_Protein,_ E_Carbohydrate_ and E_Lipid_ (expressed as mJ/mg organism). Data are reported as the mean ± SEM. No significant difference in E_a_ was observed in *L. variegatus* after CBD or 7-OH-CBD exposure when analyzed by a two-tailed *t*-test when compared with vehicle controls. Veh = vehicle control of 0.5% (v/v) dimethyl sulfoxide in artificial pond water for CBD, 0.5% (v/v) methanol in artificial pond water for 7-OH-CBD; E_a_ = total energy available; E_Protein_ = energy from protein; E_Carbohydrate_ = energy from carbohydrates; E_Lipid_ = energy from lipids.

## Discussion

In this study, we demonstrate, for the first time, that CBD induces significant effects on *L. variegatus* behavior, physiology, and regenerative capacity. We also show that the effects of the CBD metabolites, 7-OH-CBD, and 7-COOH-CBD, are lessened compared with the parental compound.

Several studies have now detected CBD in environmental samples (Black et al., 2019; Jurado et al., 2012; Mastroianni et al., 2013) but it should be noted that these studies are from limited geographical locations, namely, the United States and Spain. Levels of environmental CBD globally may vary depending on legislation on medicinal cannabis, cannabinoid-based therapies, and recreational cannabis use.

Behaviorally, when exposed to environmentally relevant concentrations of CBD (Mastroianni et al., 2013) for 24 hr, we observed dose-dependent decreases in responses to tactile stimulation to elicit stereotypical movements of body reversal and helical swimming, as well as locomotor activity. Conversely, CBD has induced behavioral changes in the commonly used invertebrate *Caenorhabditis elegans* (Land et al., 2021). These decreases in tactile response and locomotion are akin to previous studies observing these behaviors in *L. variegatus* (Carriere et al., 2023; Davies et al., 2025; O’Gara et al., 2004; Seeley et al., 2021, 2024). It has previously been shown that exposure to pharmacologically active compounds for 10 min can induce significant behavioral changes in *L. variegatus* (Carriere et al., 2023; Davies et al., 2025; Seeley et al., 2021, 2024). As such, we also describe the effects of *L. variegatus* exposed to short-term CBD for 10 min and we observed a rapid decrease in response to tactile stimulation that was not readily recoverable, with 5 µM CBD capable of inducing effects after just 10 min of exposure. *Lumbriculus variegatus*, when exposed to ≤ 20 µM CBD, exhibited almost total lethality at 20 µM. These observations demonstrate the increased toxicity of CBD in *L. variegatus* compared with other invertebrates. *Caenorhabditis elegans* exposed to magnitudes of CBD far in excess of those tested in our study displayed no lethality when exposed to 4,000 µM CBD for 6 hr or 100 µM for up to 5 days (Land et al., 2021). Conversely, CBD exposure was shown to increase *C. elegans* lifespan compared with control conditions (Land et al., 2021). Other invertebrates exposed to CBD, such as *Manduca sexta*, also display reduced toxicity compared with *L. variegatus*, capable of surviving exposure to 10–2,000 µM (Park et al., 2019). Although 20 µM CBD is at over 10-fold higher concentrations than those observed in environmental samples (Mastroianni et al., 2013), CBD does seem to exert its toxicity in aquatic systems within the micromolar range for both vertebrates and invertebrates. Carty et al. (2018) examined the effects of 0.25–4.0 µM CBD in zebrafish and determined a lethal concentration that killed 50% of 1.69 µM, whereas Ahmed et al. (2018) examined the effects of 3.2–12.7 µM CBD in zebrafish and observed significant decreases in zebra fish body length at ≥ 3.2 µM. Cannabidiol has previously been shown to have detrimental effects in aquatic invertebrates, with 0.1–10 µM CBD causing significant reductions in sperm fertility in the sea urchin *Strongylocentrotus purpuratus* (Schuel et al., 1987, 1991).

Reduced movements may be through inhibition of acetylcholinesterase, responsible for the degradation of acetylcholine at neuromuscular junctions, which has previously been shown to be inhibited by CBD (Puopolo et al., 2022). Cholinesterase activity has previously been documented in *L. variegatus* (Davies et al., 2025; Silva et al., 2021). Additionally, Lesiuk and Drewes (1999) and Davies et al. (2025) observed the effects of nicotine in *L. variegatus*, suggesting the presence of cholinergic motor neurons, which has been documented in other annelids (Gerschenfeld, 1973; Walker et al., 1993). Inhibition of cholinesterase enzymes may induce hyper-contraction paralysis due to reduced acetylcholine degradation resulting in overstimulation of cholinergic receptors in the body wall muscle, thereby reducing *L. variegatus* movements. Furthermore, the DBV pulse rate in *L. variegatus* is generated by peristaltic pulsation of the muscle wall (Crisp et al., 2010; Lesiuk & Drewes, 1999). As such, the decreased DBV pulse rates in *L. variegatus* exposed to CBD that we observed could be explained by hyper-contraction paralysis preventing these peristaltic pulsations through reduced body wall muscle relaxation capacity. In future studies, it would be of interest to determine whether the CBD-induced effects persist in the presence of cholinergic antagonists, such as nicotine, and to measure cholinesterase activity in *L. variegatus* exposed to CBD.

The regenerative capacity of *L. variegatus* enables asexual reproduction or recovery from injury by segmental regeneration (Martinez Acosta et al., 2021), and we observed that CBD exposure reduced the regenerative capacity of *L. variegatus* following bisection over a period of 72 HPA. This time frame has been utilized by several studies observing the regeneration of *L. variegatus* (Tellez-Garcia et al., 2021; Tweeten & Anderson, 2008). The anticancer potential of CBD, through inhibition of cell proliferation, is well documented, as reviewed by Valenti et al. (2022), and so it is perhaps unsurprising that CBD was shown to reduce regenerative capacity in these worms. Similarly, Tweeten & Anderson (2008) described inhibition of regeneration in *L. variegatus* exposed to the mitosis-blocking agents vinblastine and colchicine in timeframes used within this study. Irrespective of the mechanism, we have demonstrated that CBD reduces the regenerative capacity of *L. variegatus*, reducing recovery from injury by segmental regeneration.

In tissue homogenates of *L. variegatus* exposed to CBD for 72 hr, we observed carbohydrate levels were significantly decreased, and lipid levels were increased, but there was no effect on protein levels. Total E_a_, however, demonstrated no significant difference suggesting that the significant changes in carbohydrates and lipids were compensatory. A previous study has demonstrated that *L. variegatus* exposed to microplastics had no alteration in protein levels, but there were significant changes in lipid and carbohydrate levels (Silva et al., 2021). Silva et al. (2021) found minimal effects on total energy levels in *L. variegatus* and suggested depletion of energy reverses could be linked to activation of detoxification mechanisms, which may be similarly occurring in this study. It may be useful to examine the effects of CBD on aerobic energy production and other biomarkers as utilized in other studies of *L. variegatus* to investigate whether the alteration of energy reserves is linked to the activation of detoxification mechanisms (Martínez et al., 2021; Silva et al., 2021). The increased lipid levels we observed here may be due in part to the ability of CBD to increase levels of the endogenous cannabinoid, the fatty acid neurotransmitter anandamide (de Almeida & Devi, 2020). “Endocannabinoid-like” signaling molecules are likely to exist within *L. variegatus*, as the endocannabinoid system is phylogenetically ancient (Clarke et al., 2021) and is present in other annelid species (Kabeiseman et al., 2020; Matias et al., 2001; Salzet & Stefano, 2002).

In this study, the effects of the CBD metabolites, 7-OH-CBD, and 7-COOH-CBD, on *L. variegatus* were also examined. It should be noted that studies that have examined CBD in the environment did not investigate the presence of CBD metabolites (Black et al., 2019; Jurado et al., 2012; Mastroianni et al., 2013). In humans, 12.1% of CBD is proposed to be excreted unchanged (Ujváry & Hanuš, 2016), with 7-OH-CBD being the most excreted CBD metabolite in urine, followed by 7-COOH-CBD (Pérez-Acevedo et al., 2020). However, CBD metabolism is proposed to be subject to large interindividual variation (Ujváry & Hanuš, 2016) and therefore, the extent to which these metabolites are produced or their prevalence in the environment remains to be determined.

There is conflicting information on 7-OH-CBD, with it being reported to have reduced (Nye et al., 1985), increased (Stott et al., 2015), or been equipotent to parental CBD (Beers et al., 2021). This may be due to the diversity of molecular targets of CBD (de Almeida & Devi, 2020), and by extension, its metabolites. 7-Carboxy-cannabidiol is reported to be an inactive metabolite of CBD, being devoid of receptor affinity at several molecular targets of CBD (Ujváry & Hanuš, 2016; Zhang et al., 2024). In our study, we observed toxicity in 50% of our test populations at lower concentrations of 7-OH-CBD, but higher concentrations of 7-COOH-CBD, compared with CBD.

Following 24-hr exposure to CBD, we observed inhibition of body reversal and helical swimming at ≥ 2.5 µM and ≥ 0.5 µM, respectively. Comparatively, 7-OH-CBD inhibited these movements at 5 µM only whereas 7-COOH-CBD did not affect tactile stimulation to elicit stereotypical movements. Additionally, 24-hr exposure to 5 µM CBD significantly inhibited locomotor activity of *L. variegatus* whereas 5 µM 7-OH-CBD and 7-COOH-CBD had no significant effect on locomotion. As such, based on the metabolites we examined in this study, we demonstrate that CBD is of more pertinent concern as an environmental contaminant than the tested metabolites in this organism. However, approximately 100 CBD metabolites have been described across different organisms (Ujváry & Hanuš, 2016), and so the environmental impact of these other metabolites remains to be determined.

## Conclusion

Our study provides evidence of the negative effects of CBD on the endobenthic detritivore *Lumbriculus variegatus* and the associated decrease in behavioral, physiological, regenerative, and biochemical responses. Our results suggest that CBD in the environment could reduce *L. variegatus* capacity to avoid predation due to decreased stereotypical movements, reduced locomotor activity, and reduced regenerative capacity following injury. As detritivores, *L. variegatus* contribute to the ecosystem services of soil formation and nutrient cycling, and so detrimental effects on *L. variegatus* may affect these ecosystem services. Cannabidiol and other cannabinoids have been measured in environmental samples, and here, we show the potential detrimental effects of CBD as an environmental contaminant of one aquatic species.

## Supplementary Material

vgaf048_Supplementary_Data

## Data Availability

The data that support the findings of this study are available on reasonable request from the Swansea Worm Integrative Research Laboratory data repository. For access, contact SWIRL@swansea.ac.uk.
