# Supplementary material for: The behavioral, physiological, and biochemical responses of Lumbriculus variegatus exposed to cannabidiol and its metabolites
Source: Environ Toxicol Chem. 2025 Feb 14;44(5):1297–309. doi: 10.1093/etojnl/vgaf048 (PMC12047024; doi:10.1093/etojnl/vgaf048)
Supplement: vgaf048_Supplementary_Data [file vgaf048_supplementary_data.zip › Figure S2 Figure Legend.docx]

**Figure S2:** **The effect of 24-hour exposure to 0 – 5 µM 7-COOH-CBD on *Lumbriculus variegatus*behaviour.** *L. variegatus* were exposed to 7-COOH-CBD (0 – 5 µM) for 24 hours and tested for the ability of tactile stimulation to elicit **(A)** body reversal or **(B)**helical swimming. Following removal of 7-COOH-CBD, the ability of *L. variegatus* to perform **(C)** body reversal or **(D)** helical swimming was tested after 10 minutes and 24 hours. Data are expressed as a ratio of the movement score after exposure relative to the movement score at baseline.**(E)** Representative superimposed images analysed in ImageJ showing the effect of 24 hours of exposure to 7-COOH-CBD on locomotor activity measured before 7-COOH-CBD exposure (Baseline), after 24 hours of exposure to 0 – 5 µM 7-COOH-CBD (7-COOH-CBD Treatment (24 h)), 10 minutes after 7-COOH-CBD removal (Recovery (10mins)) and 24 hours after 7-COOH-CBD removal (Recovery (24h)). Quantification of the area covered by*L. variegatus* following **(F)**10 minutes of exposure to 0 – 5 µM 7-COOH-CBD and**(G)** removal of 7-COOH-CBD for 10 minutes and 24 hours are expressed as a percentage of the locomotor activity at baseline. Analyses were conducted by comparing 7-COOH-CBD exposure conditions to baseline conditions by paired non-parametric two-tailed *t*-test for stereotypical movement assays and paired parametric two-tailed *t*-test for locomotor activity. A two-way ANOVA with Dunnett’s post-test was used to analyse 10-minute and 24-hour recovery time points compared to baseline conditions for *L. variegatus*. */# *p*<.05 where * refers to statistical significance between Baseline and CBD exposure (24 h), # refers to statistical significance between Baseline and Recovery (24 h). Error bars represent the standard error of the mean, *n*=8 with a single *L. variegatus* exposed to each concentration. Veh = 0.5 % (v/v) methanol in artificial pond water; 7-COOH-CBD = 7-carboxy-cannabidiol.
