# Supplementary figures and images for: The behavioral, physiological, and biochemical responses of Lumbriculus variegatus exposed to cannabidiol and its metabolites
Source: Environ Toxicol Chem. 2025 Feb 14;44(5):1297–309. doi: 10.1093/etojnl/vgaf048 (PMC12047024; doi:10.1093/etojnl/vgaf048)

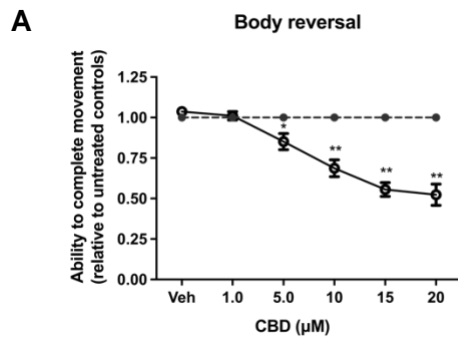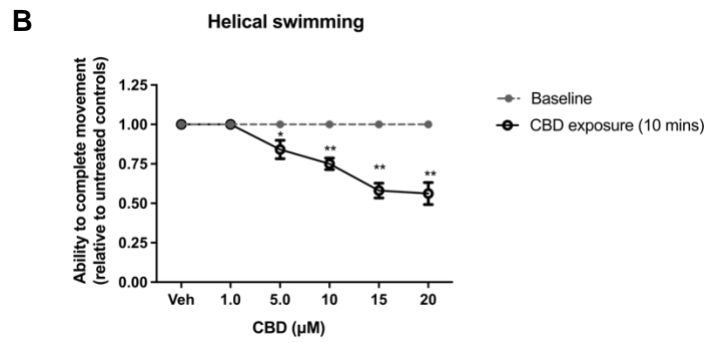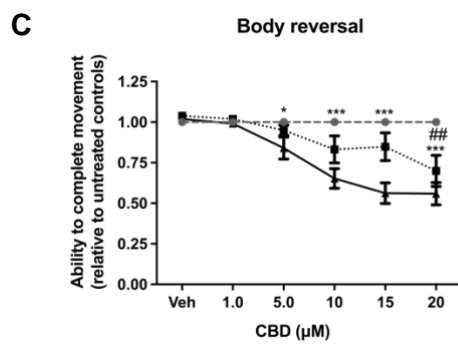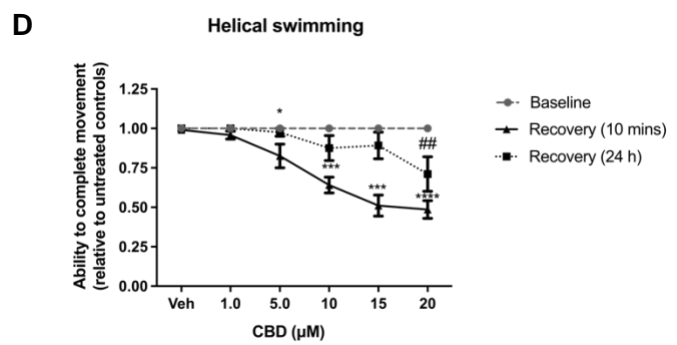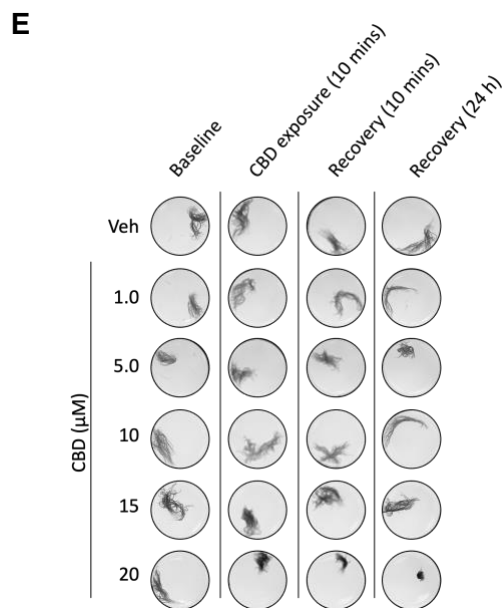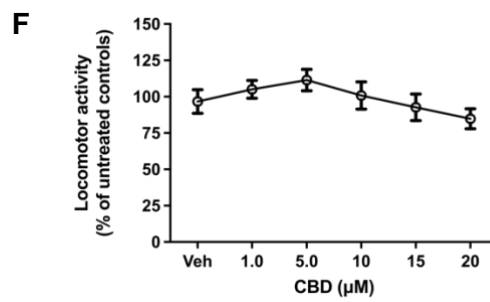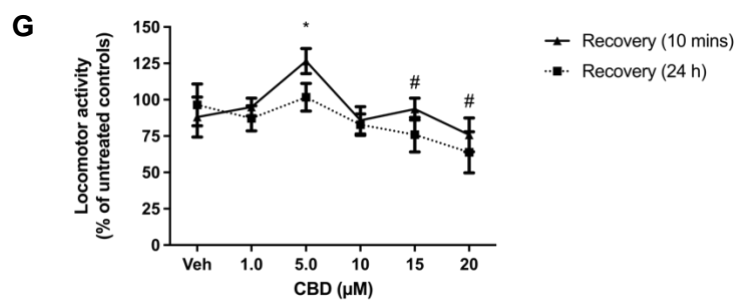

Supplement: vgaf048_Supplementary_Data [file vgaf048_supplementary_data.zip › Figure S1.pdf]

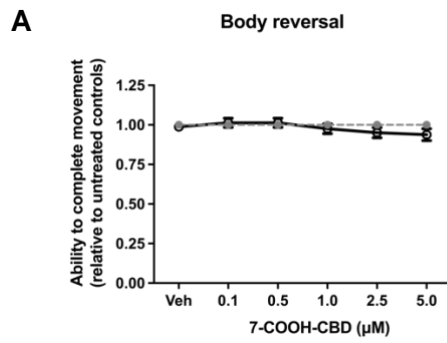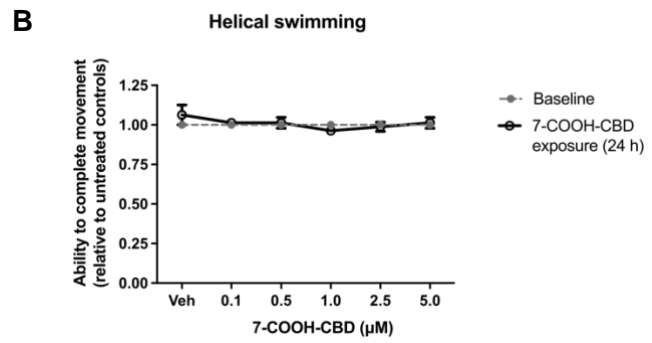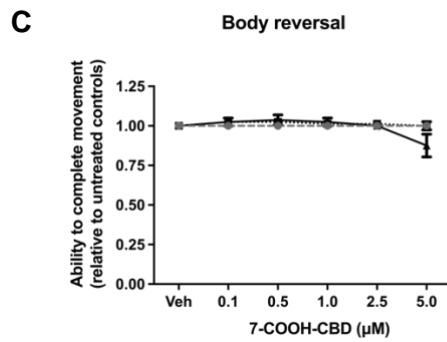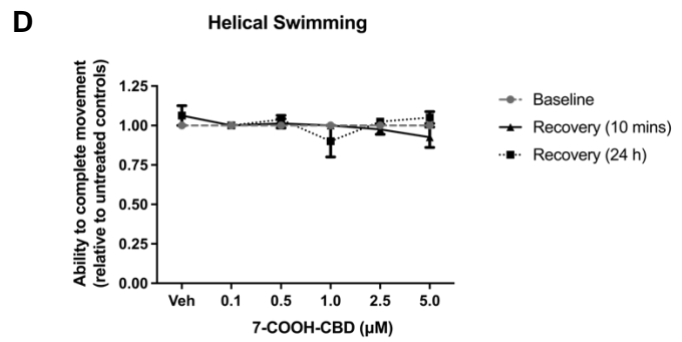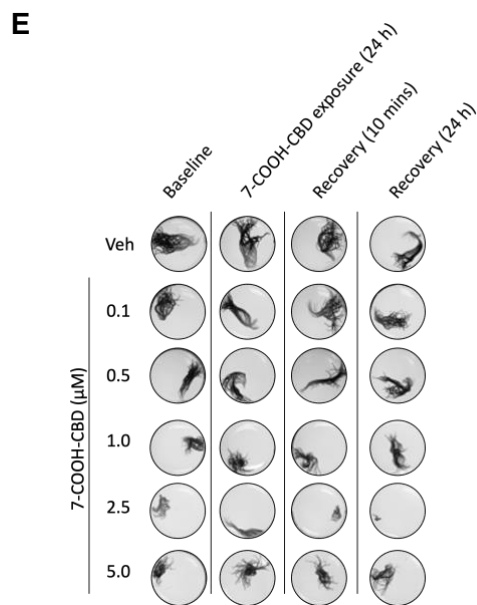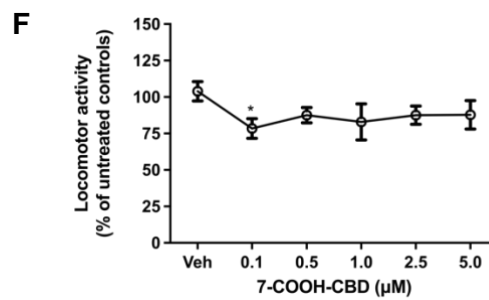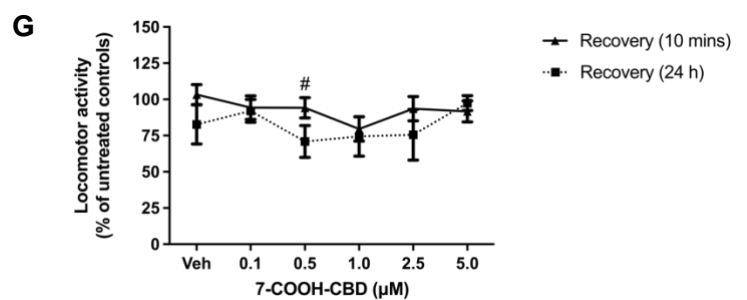

Supplement: vgaf048_Supplementary_Data [file vgaf048_supplementary_data.zip › Figure S2.pdf]
